# Supplementary material for: Element analysis: a wavelet-based method for analysing time-localized events in noisy time series
Source: Proc Math Phys Eng Sci. 2017 Apr 26;473(2200):20160776. doi: 10.1098/rspa.2016.0776 (PMC5415685; doi:10.1098/rspa.2016.0776)
Supplement: Supplementary text [file rspa20160776supp1.pdf]

Supplementary text for “Element analysis” by J. M. Lilly

Section S1. Moments and cumulants of generalized Morse wavelets

The frequency-domain *moments* of the generalized Morse wavelets, which are utilized several times in the main text, are defined as [see 18, § III-A]

$$M_{n;\beta,\gamma} \equiv \frac{1}{2\pi}\int_0^\infty \omega^n \Psi_{\beta,\gamma}(\omega)\mathrm{d}\omega = \frac{a_{\beta,\gamma}}{2\pi}\int_0^\infty \omega^{\beta+n}\mathrm{e}^{-\omega^\gamma}\mathrm{d}\omega = \frac{a_{\beta,\gamma}}{2\pi\gamma}\Gamma\left(\frac{\beta+1+n}{\gamma}\right) \tag{S 1}$$

where the last expression follows from the change of variables $u=\omega^\gamma$ together with the definition of the gamma function $\Gamma(x)\equiv\int_0^\infty u^{x-1}\mathrm{e}^{-u}\mathrm{d}u$. The moments are the terms in the Taylor series

$$\psi_{\beta,\gamma}(t) = \sum_{n=0}^{\infty}\frac{(\mathrm{i}t)^n}{n!}M_{n;\beta,\gamma} \tag{S 2}$$

from which we see that the zeroth-order moment is the value of the wavelet at its temporal center,

$$\psi_{\beta,\gamma}(0) = \frac{1}{2\pi}\int_0^\infty \Psi_{\beta,\gamma}(\omega)\mathrm{d}\omega = M_{0;\beta,\gamma} = \frac{a_{\beta,\gamma}}{2\pi\gamma}\Gamma\left(\frac{\beta+1}{\gamma}\right). \tag{S 3}$$

This expression is used in setting the values of the coefficients $|c_n|$ in the element model. In the synthetic example of figure 3, we choose $|c_n\psi_{1,2}(0)|=2$. From $a_{\beta,\gamma}=2\,(\mathrm{e}\gamma/\beta)^{\beta/\gamma}$ we have $a_{1,2}=2\sqrt{2\mathrm{e}}=4.66$, and then $\psi_{1,2}(0)=4.66/(4\pi)=0.37$, leading to $|c_n|=5.39$ as stated in § 3(e).

Closely related to the wavelet moments are the wavelet *cumulants*, which are the coefficients in the expansion of the natural logarithm of the wavelet, such that the wavelet is given by

$$\psi_{\beta,\gamma}(t) = \exp\left\{\sum_{n=0}^{\infty}\frac{(\mathrm{i}t)^n}{n!}K_{n;\beta,\gamma}\right\}. \tag{S 4}$$

Equating the wavelet's moment and cumulant expansions, we find

$$\mathrm{e}^{K_{0;\beta,\gamma}}\left[1+\mathrm{i}K_{1;\beta,\gamma}t-\frac{1}{2}\left(K_{2;\beta,\gamma}+K_{1;\beta,\gamma}^2\right)t^2+\ldots\right] = M_{0;\beta,\gamma}\left[1+\mathrm{i}\frac{M_{1;\beta,\gamma}}{M_{0;\beta,\gamma}}t-\frac{1}{2}\frac{M_{2;\beta,\gamma}}{M_{0;\beta,\gamma}}t^2+\ldots\right] \tag{S 5}$$

from which we have omitted powers of t higher than third order. Equating powers of t then leads to $M_{0;\beta,\gamma}=\mathrm{e}^{K_{0;\beta,\gamma}}$ together with

$$K_{1;\beta,\gamma}\equiv\frac{M_{1;\beta,\gamma}}{M_{0;\beta,\gamma}}, \qquad K_{2;\beta,\gamma}\equiv\frac{M_{2;\beta,\gamma}}{M_{0;\beta,\gamma}}-\frac{M_{1;\beta,\gamma}^2}{M_{0;\beta,\gamma}^2}. \tag{S 6}$$

Equation (4.18) in the main text follows from this expression together with (S 1) for the moments.

Section S2. The transform of a Morse function

In this section, the expression (3.5) for the Morse wavelet transform of another Morse function is derived. Here we define $\zeta_{\beta,\mu,\gamma}(\tau,s)$ as the transform of a Morse function with $\rho=1$, and find

$$\begin{aligned}\zeta_{\beta,\mu,\gamma}(\tau,s) &\equiv \int_{-\infty}^{\infty}\frac{1}{s}\psi^{*}_{\beta,\gamma}\left(\frac{t-\tau}{s}\right)\psi_{\mu,\gamma}(t)\,\mathrm{d}t\\ &= \frac{a_{\beta,\gamma}\,a_{\mu,\gamma}}{(2\pi)^2}\int_0^\infty\int_0^\infty\int_{-\infty}^{\infty}(s\omega)^\beta\nu^\mu \mathrm{e}^{-(s\omega)^\gamma}\mathrm{e}^{-\nu^\gamma}\mathrm{e}^{\mathrm{i}\omega\tau-\mathrm{i}(\omega-\nu)t}\,\mathrm{d}t\,\mathrm{d}\omega\,\mathrm{d}\nu\\ &= \frac{a_{\beta,\gamma}\,a_{\mu,\gamma}}{2\pi}\int_0^\infty\int_0^\infty(s\omega)^\beta\nu^\mu \mathrm{e}^{-(s\omega)^\gamma}\mathrm{e}^{-\nu^\gamma}\mathrm{e}^{\mathrm{i}\omega\tau}\delta(\omega-\nu)\,\mathrm{d}\omega\,\mathrm{d}\nu\\ &= \frac{a_{\beta,\gamma}\,a_{\mu,\gamma}}{2\pi}\int_0^\infty(s\omega)^\beta\omega^\mu \mathrm{e}^{-(s\omega)^\gamma}\mathrm{e}^{-\omega^\gamma}\mathrm{e}^{\mathrm{i}\omega\tau}\,\mathrm{d}\omega\\ &= \frac{a_{\beta,\gamma}\,a_{\mu,\gamma}}{2\pi}\frac{s^\beta}{\left(\sqrt[\gamma]{s^\gamma+1}\right)^{\beta+\mu}}\int_0^\infty\left(\sqrt[\gamma]{s^\gamma+1}\,\omega\right)^{\beta+\mu}\mathrm{e}^{-\left(\sqrt[\gamma]{s^\gamma+1}\,\omega\right)^\gamma}\mathrm{e}^{\mathrm{i}\omega\tau}\,\mathrm{d}\omega\end{aligned}\qquad(\mathrm{S}\,7)$$

after substituting the wavelet definition (2.1) to obtain the second line, and where $\delta(\omega)$ is again the Dirac delta function. However the wavelet $\psi_{\beta+\mu,\gamma}(t)$ itself can be rescaled to give

$$\frac{1}{\sqrt[\gamma]{s^\gamma+1}}\,\psi_{\beta+\mu,\gamma}\left(\frac{\tau}{\sqrt[\gamma]{s^\gamma+1}}\right)=\frac{a_{\beta+\mu}}{2\pi}\int_0^\infty\left(\sqrt[\gamma]{s^\gamma+1}\,\omega\right)^{\beta+\mu}\mathrm{e}^{-\left(\sqrt[\gamma]{s^\gamma+1}\,\omega\right)^\gamma}\mathrm{e}^{\mathrm{i}\omega\tau}\mathrm{d}\omega\qquad(\mathrm{S}\,8)$$

as follows from the wavelet definition (2.1). Combining the previous two expressions gives (3.5), as claimed. Then (3.4) follows from a change of variables.

Section S3. The wavelet spectrum of power-law noise

In this section we derive (4.7) for the expected value of the magnitude-squared wavelet transform, or wavelet spectrum, of power-law noise. To do so we will express the wavelet spectrum of noise in terms of a wavelet moment. The wavelet spectrum of noise (4.5) is found to be

$$\mathrm{E}\left\{\left|\varepsilon_{\beta,\gamma}(\tau,s)\right|^2\right\}=\frac{1}{2\pi}\int_0^\infty\Psi^2_{\beta,\gamma}(s\omega)A^2\omega^{-2\alpha}\mathrm{d}\omega=A^2s^{2\alpha-1}f_{\alpha,\beta,\gamma}\qquad(\mathrm{S}\,9)$$

after employing a change of variables, and with $f_{\alpha,\beta,\gamma}$ given by

$$\begin{aligned}f_{\alpha,\beta,\gamma}&\equiv\frac{1}{2\pi}\int_0^\infty\omega^{-2\alpha}\,\Psi^2_{\beta,\gamma}(\omega)\mathrm{d}\omega=\frac{a^2_{\beta,\gamma}}{2\pi}\int_0^\infty\omega^{2\beta-2\alpha}\mathrm{e}^{-2\omega^\gamma}\mathrm{d}\omega\\ &=\frac{a^2_{\beta,\gamma}}{2^{(2\beta-2\alpha+1)/\gamma}}\frac{1}{2\pi}\int_0^\infty\omega^{2\beta-2\alpha}\mathrm{e}^{-\omega^\gamma}\mathrm{d}\omega=\frac{a^2_{\beta,\gamma}}{2\pi\gamma}\frac{\Gamma\left(\frac{2\beta-2\alpha+1}{\gamma}\right)}{2^{(2\beta-2\alpha+1)/\gamma}}\end{aligned}\qquad(\mathrm{S}\,10)$$

with the second line following from a second change of variables $\omega\mapsto\omega/2^{1/\gamma}$, and then using the definition of the gamma function as in (S 1). Observe that in order for the integral defining $f_{\alpha,\beta,\gamma}$ to evaluate to a finite value, we must have $\beta>\alpha-\frac{1}{2}$, which keeps the argument of the gamma function in (4.8) positive. Intuitively this means that the decay of the wavelet toward zero frequency, controlled by β, must be strong enough to overcome the singularity in the Fourier spectrum of the noise $S_\epsilon(\omega)$, resulting in an integrable singularity weaker than ω^{-1}.

The moments of the *squared* wavelet, referred to as the *energy moments* by [18], are defined as [see § III-A of 18]

$$N_{n;\beta,\gamma}\equiv\frac{1}{2\pi}\int_0^\infty\omega^n\Psi^2_{\beta,\gamma}(\omega)\mathrm{d}\omega=\frac{a^2_{\beta,\gamma}}{2\pi}\int_0^\infty\omega^{2\beta+n}\mathrm{e}^{-2\omega^\gamma}\mathrm{d}\omega\qquad(\mathrm{S}\,11)$$

so that $f_{\alpha,\beta,\gamma}$ can alternately be expressed in terms of the energy moments as $f_{\alpha,\beta,\gamma}=N_{-2\alpha;\beta,\gamma}$. This is how $f_{\alpha,\beta,\gamma}$ is implemented in the `jLab` toolbox.

Section S4. The wavelet transform autocovariance function

In this appendix, we derive (4.13) expressing the normalized wavelet transform autocovariance function $\Xi_{\alpha,\beta,\gamma}(u,s,r)$ in terms of a wavelet. To show this, we substitute the frequency-domain form of the wavelet transform (2.4) into the definition (4.12), giving

$$\Xi_{\alpha,\beta,\gamma}(u,s,r) = \frac{1}{2\pi}\int_0^\infty \mathrm{e}^{-\mathrm{i}\omega u}\Psi_{\beta,\gamma}(s\omega)\Psi_{\beta,\gamma}(rs\omega)S_\epsilon(\omega)\mathrm{d}\omega \tag{S 12}$$

after employing the definition of the noise spectrum (4.2). Inserting the form of the power-law noise spectrum $S_\epsilon(\omega) = A^2\omega^{-2\alpha}$, this expression becomes

$$\Xi_{\alpha,\beta,\gamma}(u,s,r) = A^2 a_{\beta,\gamma}^2 \frac{1}{2\pi}\int_0^\infty (s\omega)^\beta (rs\omega)^\beta \omega^{-2\alpha}\mathrm{e}^{-(s\omega)^\gamma-(rs\omega)^\gamma}\mathrm{e}^{-\mathrm{i}\omega u}\mathrm{d}\omega \tag{S 13}$$

using the wavelet definition (2.1). In terms of $\tilde{r}_\gamma \equiv \sqrt[\gamma]{1+r^\gamma}$ this may be rewritten as

$$\Xi_{\alpha,\beta,\gamma}(u,s,r) = \frac{A^2 a_{\beta,\gamma}^2}{a_{2\beta-2\alpha,\gamma}}\frac{r^\beta s^{2\alpha-1}}{\tilde{r}_\gamma^{2\beta-2\alpha+1}}\left[\frac{s\tilde{r}_\gamma}{2\pi}\int_0^\infty a_{2\beta-2\alpha,\gamma}(s\tilde{r}_\gamma\omega)^{2\beta-2\alpha}\mathrm{e}^{-(s\tilde{r}_\gamma\omega)^\gamma}\mathrm{e}^{-\mathrm{i}\omega u}\mathrm{d}\omega\right] \tag{S 14}$$

however, from the wavelet scaling law

$$\psi_{\beta,\gamma}(t/s) = \frac{s}{2\pi}\int_{-\infty}^\infty \Psi_{\beta,\gamma}(s\omega)\mathrm{e}^{\mathrm{i}\omega t}\,\mathrm{d}\omega = \frac{s}{2\pi}\int_{-\infty}^\infty a_{\beta,\gamma}(s\omega)^\beta\mathrm{e}^{-(s\omega)^\gamma}\mathrm{e}^{\mathrm{i}\omega t}\,\mathrm{d}\omega \tag{S 15}$$

one sees that the quantity in brackets in (S 14) is the same as $\psi^*_{2\beta-2\alpha,\gamma}(u/(s\tilde{r}_\gamma))$, leading to

$$\Xi_{\alpha,\beta,\gamma}(u,s,r) = A^2\frac{a_{\beta,\gamma}^2}{a_{2\beta-2\alpha,\gamma}}\frac{r^\beta s^{2\alpha-1}}{\tilde{r}_\gamma^{2\beta-2\alpha+1}}\psi^*_{2\beta-2\alpha,\gamma}\left(\frac{u}{s\tilde{r}_\gamma}\right). \tag{S 16}$$

This can be simplified further by rearranging (4.7) to give $\sigma^2_{\alpha,\beta,\gamma}(s)/f_{\alpha,\beta,\gamma} = A^2 s^{2\alpha-1}$. Substituting that expression into the above leads to (4.13).

Recalling from (S 3) that the value of the $(2\beta-2\alpha,\gamma)$ wavelet at its temporal center is given by

$$\psi_{2\beta-2\alpha,\gamma}(0) = \frac{a_{2\beta-2\alpha,\gamma}}{2\pi\gamma}\Gamma\left(\frac{2\beta-2\alpha+1}{\gamma}\right) \tag{S 17}$$

one finds the following simplification for the ratio of this wavelet to the $f_{\alpha,\beta,\gamma}$ function

$$\frac{\psi_{2\beta-2\alpha,\gamma}(0)}{f_{\alpha,\beta,\gamma}} = \frac{a_{2\beta-2\alpha,\gamma}}{a_{\beta,\gamma}^2}2^{(2\beta-2\alpha+1)/\gamma}. \tag{S 18}$$

Substituting this expression into (4.13), we find $\Xi_{\alpha,\beta,\gamma}(0,s,1) = \sigma^2_{\alpha,\beta,\gamma}(s)$, as claimed in the text.
