# Supplementary material for: Element analysis: a wavelet-based method for analysing time-localized events in noisy time series
Source: Proc Math Phys Eng Sci. 2017 Apr 26;473(2200):20160776. doi: 10.1098/rspa.2016.0776 (PMC5415685; doi:10.1098/rspa.2016.0776)
Supplement: Supplementary figures [file rspa20160776supp2.pdf]

Supplementary figures for “Element analysis” by J. M. Lilly

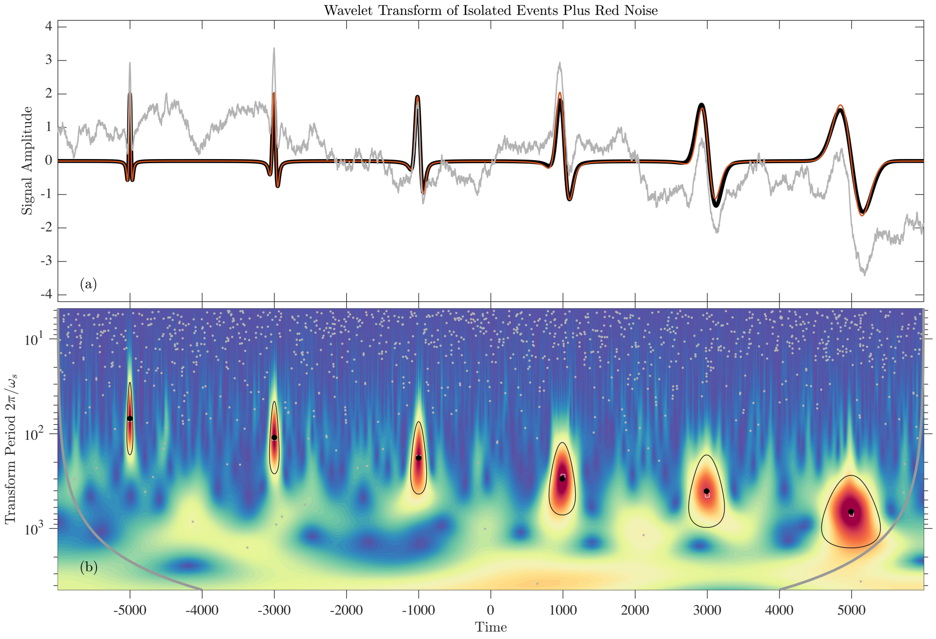

Figure S1. As with figure 3 in the main text, but with the noise consisting of a realization of $\alpha = 1$ red noise rather than white noise. The red noise is formed by cumulatively summing discrete Gaussian white noise, then setting the standard deviation to unity and removing the mean. All other aspects of this plot are identical with figure 3.

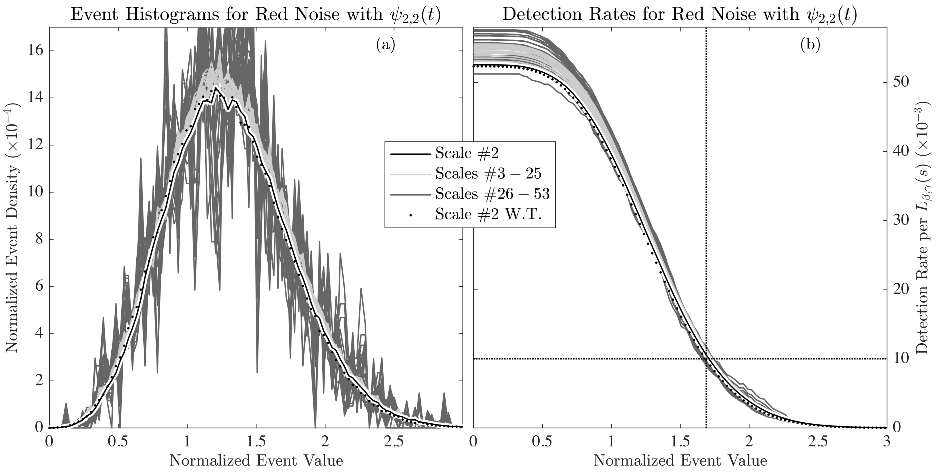

Figure S2. As with figure 5 in the main text, but for $\alpha = 1$ red noise. The differences between the normalized curves in the white and red noise cases are marginal, and are primarily limited to the magnitudes of event densities and detection rates rather than the shapes of the curves; note that the y-axes here are different from those in figure 5.

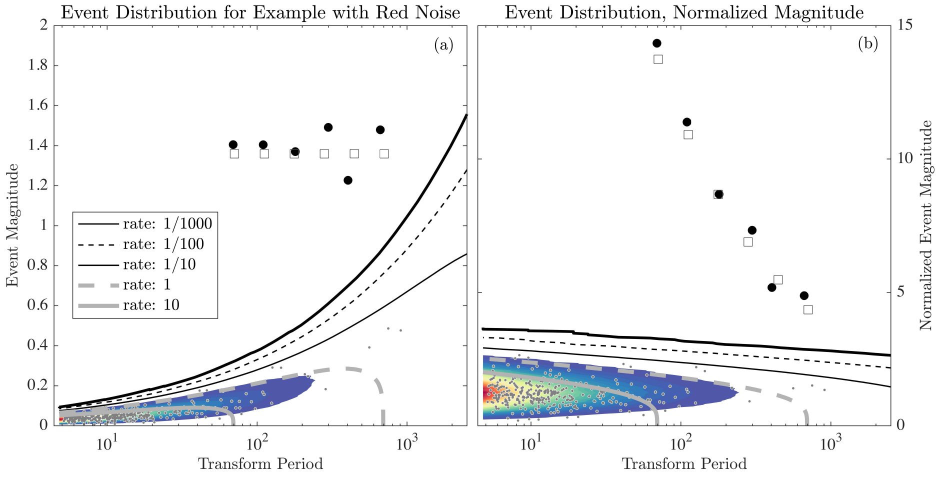

Figure S3. As with figure 6 in the main text, but for $\alpha = 1$ red noise. Unlike in the white noise case, detected event magnitudes due entirely to the noise tend to increase with increasing scale or period, as is clear from panel (a). Normalization by the wavelet spectrum of the noise, as in (b), makes the noise distributions qualitatively similar between the red and white noise cases; however, there is now the important difference that events of a fixed magnitude, such as the six large-amplitude events in the analyzed signal, becomes *less* rather than more significant as one proceeds toward larger scales or periods. This figure is used to assess the significance of events in figure S1. As with the white noise case, the six large-amplitude events are again highly statistically significant.

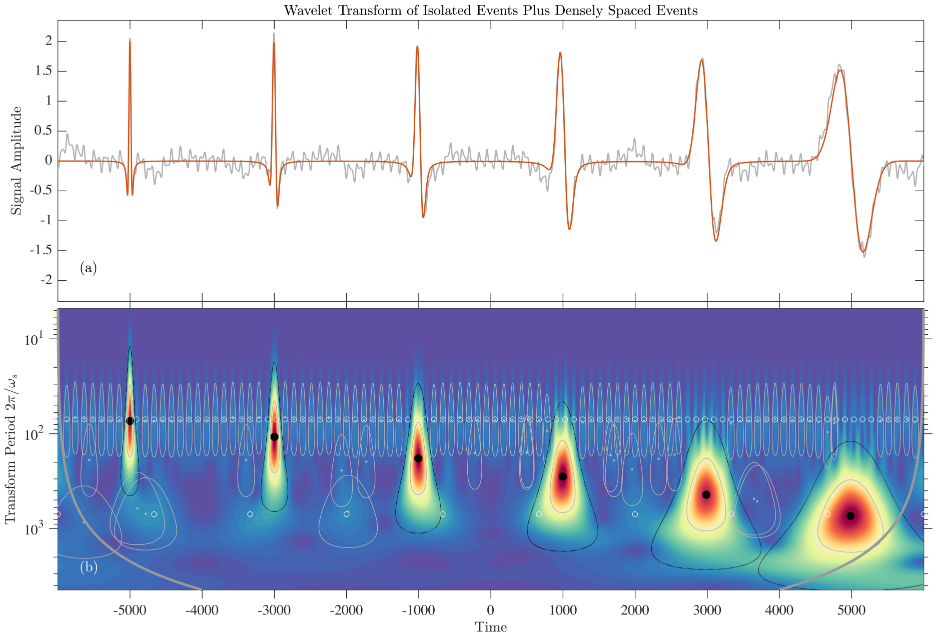

Figure S4. As with figure 3 in the main text, but for the chain of six large-amplitude events shown in that figure plus an array of closely spaced, weaker events rather than white noise. This figure explores the sensitivity of event detection to the presence of nearby, stronger events. The red line in (a) shows the signal due to the six large-amplitude events. To this is added a set of 110 smaller-amplitude events, with the total signal shown as a gray line. (Unlike figure 3, no reconstruction is shown in (a), because the focus here is on detection.) The smaller-amplitude signals, like the larger-amplitude signals, are the real parts of phase-rotated $\psi_{1,2}(t)$ wavelets. The peak magnitudes of all of these small events is set to 1/10 that of the large-amplitude events, while their phases are random. 100 of these are uniformly spaced in time with a frequency of $\omega_\rho = 2\pi/100$, while then remaining ten have the lower frequency $\omega_\rho = 2\pi/1000$ and are also uniformly spaced in time. Using (3.15), these peak frequencies are found correspond to scale frequencies within the transform of $\omega_s = \sqrt{2}\omega_\rho$, or $\omega_s = 2\pi\sqrt{2}/100$ and $\omega_s = 2\pi\sqrt{2}/1000$ respectively. The locations of the small-amplitude events are marked in (b) with the small white circles, and occur in two parallel rows corresponding to the two different scales of events. In the wavelet transform plot in (b), the detected large-amplitude events are again marked as solid black circles, while the detected small-amplitude events are marked as gray dots. Gray curves show the $\lambda = 1/2$ regions of influence, as defined in § 4(d), around each detected maximum, while black dotted lines show the larger $\lambda = 1/10$ regions of influence around just the large-amplitude maxima. The purpose of this figure is to compare the true locations of the smaller-amplitude maxima, marked by the white circles, with the inferred locations as marked by the gray dots surrounded by gray curves. It is seen that when the smaller-amplitude maxima are sufficiently distant from the large-amplitude maxima, they are accurately detected. However, those in the vicinity of the larger-amplitude maxima are obscured. As the smaller-amplitude maxima are 1/10 as strong as the larger-amplitude maxima, the obscured region extends past the location at which the larger-amplitude maxima have decayed to 1/10 of their value, marked by the dotted black curves. Spurious maxima, mostly arising from the interaction between the larger and smaller-scale layers of the small-amplitude events, are also seen. Note that some of these would be removed by the isolation criterion, which has not yet been applied in this plot. This illustrates some limitations of the element analysis method due to the interaction of nearby elements.
